# Efficacy of adjunctive intensive transcranial direct current stimulation of different cortices in treatment-resistant depression: a study protocol for a randomized double-blinded sham-controlled trial

**DOI:** 10.1186/s12888-022-04465-2

**Published:** 2022-12-19

**Authors:** Yiming Chen, Dongbin Lyu, Fan Wang, Qinte Huang, Weichieh Yang, Mengke Zhang, Zheyi Wei, Shuxiang Shi, Shuqi Kong, Shentse Chen, Shuang He, Vivien Yang, Yiru Fang, Wu Hong

**Affiliations:** 1 Shanghai Mental Health Center, Shanghai Jiao Tong University School of Medicine, Shanghai, China; 2 Shanghai Key Laboratory of Psychotic Disorders, Shanghai, China; 3 CAS Center for Excellence in Brain Science and Intelligence Technology, Shanghai, China

**Keywords:** Treatment-resistant depression (TRD), Transcranial direct current stimulation (tDCS), Dorsolateral prefrontal cortex (DLPFC), Orbitofrontal cortex (OFC), Biomarkers, Resting-state functional magnetic resonance imaging (rs-fMRI)

## Abstract

**Background:**

Treatment-resistant depression (TRD) carries a high economic burden worldwide. Transcranial direct current stimulation (tDCS) is advantageous for improving cognition and can be safely used in the treatment of depression. The effectiveness of tDCS of the left and right orbitofrontal cortex (OFC) as adjuvant treatment in patients with TRD has rarely been explored. Therefore, the objective of this trial is to evaluate the effectiveness there of when administering left dorsolateral prefrontal cortex (DLPFC) positive stimulation or OFC negative stimulation in patients with TRD.

**Methods:**

Ninety eligible participants will be recruited to receive intervention at Shanghai Mental Health Center. Treatment will be randomly assigned in a double-blind fashion. Participants will receive either DLPFC (*n* = 30), OFC (n = 30), or sham (n = 30) tDCS, while continuing their usual pharmacotherapy at a stable dosage for at least 2 weeks before enrollment and throughout the stimulation period. All participants will receive 20 weekday stimulation sessions of 60 minutes duration each. Participants in the active group will be stimulated at 2 mA throughout the session, whereas the sham group will receive only a brief period of stimulation to mimic the sensation. After 20 stimulation sessions, no further treatment will be administered. Measurements will be conducted at regular points throughout and at 8 weeks after trial completion. The primary outcome is the change in the 17-item Hamilton Depression Rating Scale (HAMD-17) score after 20 sessions. Secondary outcomes were defined as changes in other measurement scales, cognitive function, resting-state functional magnetic resonance imaging (rs-fMRI), and serum biomarkers.

**Discussion:**

We hypothesize that, in contrast to the sham group, both the active DLPFC and OFC tDCS groups will show superiority in HAMD-17 score reduction after 5, 10, and 20 sessions. Moreover, associations of the improvement of depressive symptoms with variations in rs-fMRI and TRD-related biomarkers will be evaluated. Our study may suggest that adjunctive intensive tDCS with left DLPFC positive stimulation or right OFC negative stimulation may be effective as a novel method to relieve depressive symptoms in patients with TRD. The variation of rs-fMRI, biomarkers could be used as a potential prediction model of treatment efficacy in TRD.

**Trial registration:**

The trial protocol is registered with www.chictr.org.cn under protocol registration number ChiCTR2200058030. Date of registration: March 27, 2022. Recruitment started in September 2022 and is ongoing.

## Background

The World Health Organization ranks major depressive disorder (MDD) among the most debilitating diseases to society, partly due to its association with increased utilization of healthcare resources, diminished quality of life, and indirect personal and societal costs [1]. More than 50% of patients with MDD do not reach remission with initial treatment; of those 30–50% are unresponsive to treatment [2]. The designation “treatment-resistant depression (TRD)” is used to describe patients who do not respond to antidepressant therapy despite the use of appropriate dosages in one or more adequate trials of at least 6 weeks duration [3], which contributes to a disproportionately high burden of illness. The treatment of choice hereafter has traditionally been electroconvulsive therapy (ECT). Although effective, this method of neuromodulation can have a deleterious effect on the cognitive function of patients during the course of treatment [4].

Another promising neuromodulatory method that may be used in this scenario is transcranial direct current stimulation (tDCS). Unlike ECT, tDCS consists of the application of a low-intensity current for longer periods (typically 20–30 min) through electrodes that are applied to the scalp. Previous results have suggested that tDCS may be an effective treatment for MDD; however, its role in treating TRD remains unclear. A meta-analysis by Brunoni et al. (2016) [5] found that the likelihood of response to tDCS was positively correlated with treatment duration and dose but negatively correlated with treatment resistance (defined as a failure to respond to two previous trials of antidepressants). To date, some studies [6–10] have specifically examined the use of tDCS in patients with TRD; all were limited by their small sample sizes and relatively low-intensity protocols, among other methodological issues.

The dorsolateral prefrontal cortex (DLPFC) is the most common stimulation target for tDCS in the treatment of MDD and its antidepressant efficacy has been confirmed. To date, the orbitofrontal cortex (OFC) has been found to be a promising new target for the treatment of depression due to its role in behavioral decision-making, emotion regulation, and reward [11]. Hyperactivation of the right OFC and enhanced connectivity with other cerebral regions, which could present as suicide attempts, emotional lability, or lack of pleasure, might be functional mechanisms of TRD. Thus, targeted inhibition of the right OFC and its connectivity with other cerebral regions may be effective in TRD.

Moreover, advances in resting-state functional magnetic resonance imaging (rs-fMRI) have enabled the examination of the plasticity of large-scale networks using blood oxygen level-dependent correlations measured at rest. Resting-state functional connectivity analysis provides unique insights into experience-dependent brain plasticity in humans [12]. Analysis of cortical excitability and functional connectivity based on rs-fMRI, combined with clinical characteristics and TRD-related biomarkers, could provide a predictive model of DLPFC-tDCS or OFC-tDCS, and thus, could contribute to a targeted and individualized treatment plan for patients with TRD.

## Methods

### Objectives

The primary objective of this study is to investigate the efficacy of a 4-week treatment of intensive adjunctive tDCS with left DLPFC positive stimulation or right OFC negative stimulation on depressive symptoms as measured by the 17-item Hamilton Depression Rating Scale (HAMD-17).

The secondary objectives are as follows:

1. To investigate the effects of adjunctive treatment of TRD with intensive tDCS on cognition, measured by the Wisconsin card sorting test (WCST) and Stroop color–word test (SCWT).
2. To investigate the effects of adjunctive treatment of TRD with intensive tDCS on variations in nerve growth factor (NGF), brain-derived neurotrophic factor (BDNF), glial cell line-derived neurotrophic factor (GDNF), interleukins (IL), tumor necrosis factor-alpha (TNF-α), and its soluble receptors sTNFr1 and sTNFr2 in blood plasma.
3. To investigate the effects of combined treatment of TRD with intensive tDCS on the variation of neuronal activity in the cerebral cortex as measured by rs-fMRI.

### Trial design

**Fig. 1:**
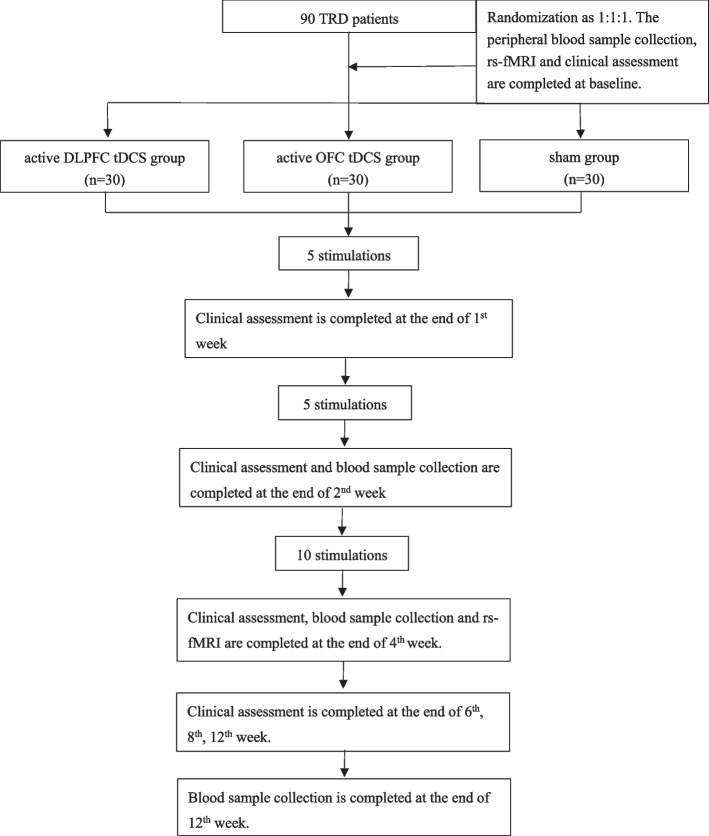
Trial flowchart. We plan to recruit 90 patients with TRD. Participants are required to continue maintenance pharmacotherapy from at least 2 weeks before stimulation intiation and throughout the stimulation period

We have adopted a randomized allocation design to test sham-controlled antidepressant augmentation. Patients with TRD will continue maintenance pharmacotherapy for at least 2 weeks before stimulation initiation and during the entire stimulation period. Groups will be randomized through random code generator software (www.randomization.com) at a ratio of 1:1:1 into one of the following three groups: active DLPFC tDCS group, active OFC tDCS group, or sham tDCS group. The total study duration will be 12 weeks (Fig. 1).

### Recruitment

We will approach patients in the Shanghai Mental Health Center outpatient and inpatient units. Our inpatient and outpatient care units follow up at least 10,000 patients with MDD each year, 20% of which are treatment-resistant. Considering the inclusion and exclusion criteria, we expect to approach 100 patients to reach the recruitment target of 90 patients. Usual clinical care will be maintained for all participants following the completion of the trial at our care unit. Recruitment, pre-participation screening, allocation, intervention, and data collection will be conducted between September 2022 and March 2024. We plan to conclude the study and have the results available for analysis at the end of 2024. The present study protocol complies with the SPIRIT 2013 recommendations (Standard Protocol Items: Recommendations for International Trials) [13]. Figure 1 shows the schedule of enrollment, interventions, and assessments.

### Eligibility criteria

#### Inclusion criteria

1. Ability and willingness to provide written informed consent to participate in the trial and undergo all necessary procedures;
2. Age from 18 to 65 years;
3. A diagnosis of MDD according to the Diagnostic and Statistical Manual of Mental Disorders, Fifth Edition (DSM-5), which is assessed by at least one professional psychiatrist;
4. Han ethnicity;
5. Right-handedness;
6. Adequate auditory and visual ability to complete the necessary checks;
7. A HAMD-17 score ≥ 17 [14];
8. Insufficient response (response rate < 50%, and a reductive ratio of HAMD-17 < 50%) to two antidepressants with different mechanisms of action for at least 6 weeks at an adequate dose (e.g., clomipramine ≥150 mg/day, fluoxetine ≥20 mg/day) during the current episode;
9. Willing to remain on maintenance pharmacotherapy for at least 2 weeks before the stimulation initiation and during the total stimulation period.

#### Exclusion criteria

1. The presence of severe systemic illnesses including, but not limited to severe hepatic, renal, endocrine, cardiovascular, respiratory, hematological or oncological diseases, deemed by the investigator to be unsuitable to allow participation in the trial;
2. Any clinically significant abnormal laboratory examination that may influence the health of participants in the opinion of the investigator;
3. A history of any significant medical illness such as neurological disorders (e.g.: cerebral trauma, seizure disorder, etc), which in the opinion of the investigator would preclude safe participation in the trial;
4. Known with current psychosis as determined by the DSM-5; or a history of a non-mood psychotic disorder;
5. Current alcohol and/or any illicit or legal drug abuse;
6. Current or planned pregnancy and/or lactation during the trial;
7. An abnormal scalp such as open wounds;
8. A score = 4 for item 3 (suicide) on the HAMD-17;
9. Having had or currently receiving modified electroconvulsive therapy (MECT) or repetitive transcranial magnetic stimulation (rTMS) in the past 1 month;
10. Participation in another clinical trial concurrently or less than 1 month prior to randomisation.

### Interventions

Participants will receive one of three interventions: active DLPFC tDCS, active OFC tDCS, and sham tDCS. tDCS-CT devices (Neuroelectrics, Starstim 8, USA) will be used for brain stimulation. The anode and cathode electrodes will be inserted in saline-soaked sponges with a diameter of 3.2 cm and then positioned over the left and right DLPFC and OFC simultaneously while using a specific headgear [15] to maintain the double blind. The current will pass through the anode and cathode corresponding to the left and right DLPFC in the active DLPFC tDCS group and the left and right OFC in the active OFC tDCS group, respectively. For the sham group, the current will be rapidly increased up to 2 mA over the first 30 s then rapidly ramped down to 0 mA over the next 30 s automatically to allow participants to feel the typical initial sensations of active tDCS. At each session, a 2 mA current will be applied for a duration of 60 min. Participants will receive a total of 20 sessions, which will be conducted daily on weekdays. Trained psychiatrists and psychologists who will be blinded to the group assignments, will administer the tDCS regimen. After completing the 20 sessions, no additional stimulation treatment will be administered.

### Discontinuation or modifications

Participants can leave the study at any time for any of the following reasons:

1. If participants do not meet the inclusion criteria;
2. If participants or their guardians decide to leave the study;
3. If participants develop a serious physical disease whether or not it is related to tDCS;
4. If participants stop taking their medication for three consecutive days, stop effective contraception, or become pregnant;
5. If participants use other treatments (including MECT, rTMS);
6. If other conditions occur, and the investigator decides to terminate the study;
7. If the participant fails to comply with the study procedures.

### Outcomes

Primary outcome: The reductive ratio of HAMD-17 after 4 weeks, as well as after 20 sessions.

Secondary outcomes: The changes in other clinical assessments such as the Young Mania Rating Scale (YMRS), Clinical Global Impression Scale (CGI), cognitive function, biomarkers representing potential mechanisms associated with TRD, and neuronal activity measured using rs-fMRI.

### Sample size

The sample size was calculated based on the data from O’Doherty et al. (2001) [11] and Cieslik et al. (2013) [16]. In these studies, active tDCS was assumed to be superior to sham tDCS in controlling depression with a conservative mean difference of 0.65 and standard deviation (SD) = 0.25. We set a type 1 error α (significance) and a power of 80%. The G power software (V3.1.9.4) was used to calculate the sample size. The results indicated a minimum requirement of 24 participants per group (a total of 72 participants). Accounting for a 20% dropout rate, 87 participants are required for our experiment. Finally, we plan to enroll 30 participants per group for a total of 90 participants.

### Recruitment

A well-trained group will be involved in the participant enrollment process, including one senior psychiatrist, one PhD student, one MD student, eight master’s students working full-time, and ten psychiatrists who referred patients for scientific research. Participants and their guardians will be provided with a thorough explanation to ensure that they fully understand the study.

The Shanghai Mental Health Center is one of the two largest mental health facilities in China, with approximately 600,000 outpatients every year and 1300 beds. We will be sure to complete the study schedule using the current protocol.

### Treatment allocation

Patients were randomly assigned to one of three groups: the active DLPFC tDCS group, active OFC tDCS group, or sham tDCS group based on a computer-generated randomization scheme (REDCap) stratified by the study staff and assigned as groups A, B, or C. Participants will not be able to accurately guess what treatment they would receive. The clinician scales are to be administered by trained and blind study staff to ensure consistency. Moreover, the internal consistency of all psychiatrists involved in the scale ratings will be checked to ensure a kappa value of 0.89.

Two senior psychiatrists familiar with the DSM-5 criteria, will guarantee an accurate diagnosis. All patients who fulfill the inclusion criteria will be randomized. Randomization will be requested by psychiatrists responsible for recruitment and clinical interviews.

### Blinding

The staff responsible for the recruitment and symptom evaluation will be blinded to the information regarding the group allocation. A staff member outside the research group will feed the data into a computer.

### Data collection methods

#### Primary study parameter/endpoint

*HAMD-17*: The primary outcome criterion is the reductive ratio of the HAMD-17 score after one, two and 4 weeks of treatment. Treatment response is defined as a 50% reduction in the HAMD-17 score, and remission as a 75% reduction at any point during the 12-week trial compared with the baseline.

#### Secondary study parameters/endpoints

1. Mini International Neuropsychiatric Interview (MINI);
2. Montgomery–Asberg Depression Rating Scale (MADRS) and Oxford Depression Questionnaire (ODQ);
3. YMRS;
4. Clinical Global Impression Scale (CGI);
5. Sheehan Disability Scale (SDS);
6. WCST and SCWT;
7. Peripheral venous blood: Biomarkers representing the inflammatory mechanisms associated with TRD will be assessed. Peripheral blood serum are to be collected and stored at − 80 °C after centrifugation. We will assess the NGF, BDNF, GDNF, IL, TNF-α, sTNFr1, and sTNFr2 levels. Serum NLR, MLR, and PLR using ELISA.
8. rs-fMRI: Patients will also receive two rs-fMRI scan sessions before the start of treatment and after 20 stimulation sessions. A rs-fMRI scan was then performed. A diffusion tensor imaging (DTI) scan with two rs-fMRI tasks was performed to assess connectivity changes after treatment.

### Participant timeline

All time points for the physical examinations, scale assessments, medication use inventories, and laboratory examinations are shown in Table 1.

**Table 1:**
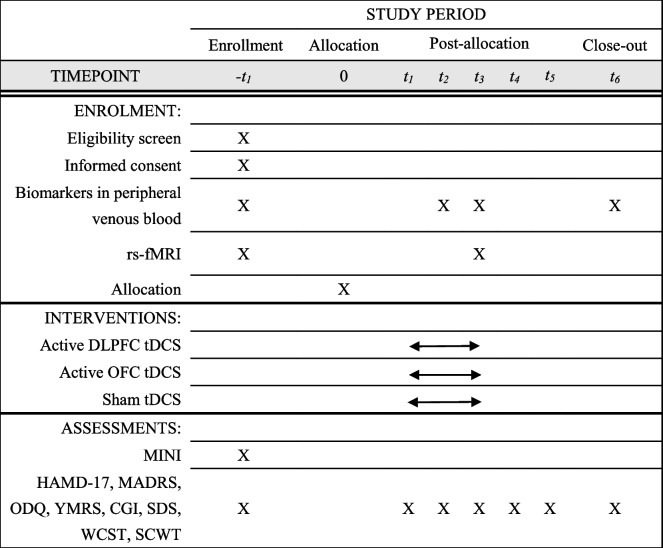
Data collection methods and clinical assessment time points

### Data management

In the tDCS study, all data will be entered electronically. All data management work will be conducted at Shanghai Mental Health Center. A special database will be built to record all data related to the tDCS study. A complete backup of the primary database will be performed once per month.

### Data monitoring

A Data Monitoring Committee (DMC) has been established. The DMC is independent of the study organizers. Interim analyses of the primary endpoint when 30 and 70% of patients have been randomised and have completed the 12-week follow-up, respectively, will be performed by an independent statistician, who will report independently to the DMC.

### Statistical methods

Descriptive analyses will consist of means and SD for continuous variables and percentages for categorical variables. Data normality will be tested with the results expressed as mean ± SD or percentage, whenever appropriate. Nonparametric tests are to be used if the data are not normally distributed. Baseline variables will be compared between the groups using either the Student’s t-test or chi-squared test (e.g., sociodemographic data, cognitive function data, biomarkers representing inflammatory mechanisms associated with TRD, and response and remission rates among groups). To compare the primary endpoint, a two-way ANOVA will be used, considering the group (intervention and sham control groups) and time points (pre- and post-intervention), followed by the Bonferroni post hoc test, in case of significant F-ratios. A mixed-effects model for repeated measures will analyze changes from baseline HAMD-17 scores, time, and their interaction. Pretreatment values (e.g., age, sex, baseline HAMD-17, and biomarker levels) will be adopted as covariates to correct for possible differences. An intention-to-treat analysis will be conducted, that is, all participants will be included in the final analysis regardless of adherence to the intervention. No additional analyses will be performed for this study. All calculations are to be performed using R software (version 4.1.3), and the significance level is set at *p* ≤ 0.05.

### Harms

All side effects for each participant will be recorded and reported throughout the study using a serious adverse event reporting system. It is unlikely that the participants in this trial will experience serious adverse events (SAEs) or serious adverse reactions. Common adverse reactions include dizziness, headache, and tingling of the scalp. Only unexpected serious adverse events or reactions unrelated to these clinical procedures will be reported as SAEs.

### Auditing

The principal investigator (PI) WH will be responsible for the ongoing management of the study. The sponsor will monitor and conduct audits on a selection of studies in its clinical research portfolio. Monitoring and auditing will be conducted in accordance with the sponsor’s monitoring and audit procedures.

### Ethics and dissemination

#### Research ethics approval

This study has been approved by the ethics committee of Shanghai Mental Health Center at Shanghai Jiao Tong University (Ethics approval number: 2021-73).

#### Protocol amendments

Any modifications to the protocol will require a formal amendment.

#### Consent or assent

Trained research psychiatrists will introduce the trial and information sheets to potential participants. Research psychiatrists will obtain written informed consent from patients or their carers. Additional serum samples and rs-fMRI data will be stored for future studies. Informed consent will obtained prior the collection of these samples.

#### Confidentiality

All laboratory specimens and data reports will only be accessed using coded ID numbers. All records that contain names or other personal identifiers will be stored separately from the study records.

#### Declaration of interests

The authors declare that they have no competing interests.

#### Access to data

All study-related and participant information will be stored in locked filing cabinets in areas with limited access. All local databases will require password access. The PI will be provided access to the cleaned datasets. All data will be password protected. To ensure confidentiality, the project team members will be blinded to the data.

#### Ancillary and post-trial care

The Shanghai Mental Health Center has insurance that covers all non-negligible harm associated with the protocol. Incidences from negligence (e.g., major protocol violations) will not be covered by the study insurance. Two weeks prior to and throughout the study period, participants will be required to continue maintenance pharmacotherapy.

#### Dissemination policy

The PI will review all publications prior to dissemination. The PI will be considered the lead author of this study. No later than 5 years after the end of recruitment, will the entire deidentified dataset be delivered to an appropriate data archive.

## Discussion

In this randomized double-blinded sham-controlled trial, we aim to determine the effectiveness of adjunctive intensive tDCS with positive stimulation of the left DLPFC or negative stimulation of the OFC on depressive symptoms in patients with TRD. In addition to their usual treatment, participants will be randomly assigned to receive either 20 weekday sessions of active DLPFC tDCS, active OFC tDCS, or sham tDCS as adjunctive treatment. We will regularly assess depression severity and functional impact using the HAMD-17, MADRS, ODQ, YMRS, CGI, and SDS throughout the trial. We will assess cognitive changes using the WCST and SCWT. Moreover, participants will be regularly assessed for treatment-related side effects using validated scales.

As OFC tDCS could have benefits in behavioral decision making and negative reinforcement; and DLPFC tDCS might improve memory, underload syndrome, and interest declination; the study will examine how depressive symptoms change in participants with TRD treated by DLPFC or OFC tDCS throughout the trial period. Entry and exit interviews will be recorded and analyzed by the staff of this research team at the Shanghai Mental Health Center.

This study has numerous strengths and will follow the Consolidated Standards of Reporting Trials (CONSORT) 2010 statement on design and reporting [17]. Using REDCap to randomly assign participants to treatment groups will ensure that the allocation is concealed, documented, and unchangeable. To minimize the placebo effect, both the participants and the treating investigators will be blinded. Primary and secondary outcomes were chosen to capture a range of subjective, objective, and functional measurements of depression severity, and the regular use of adverse event scales will ensure that the treatment side effects will be adequately captured. Measurements will also be taken at multiple points during and after the trial to better describe the overall course and persistence of treatment-related effects.

This study has several practical limitations that may affect the results. With regard to the treatment modality, delivering tDCS in a hospital setting will impose a minimal burden on inpatients; however, outpatients will have to be functional enough to use transport and to attend the hospital on a daily basis. Subsequently, there is a risk that the study population may miss outpatients with severe depression. The need for daily travel to a hospital can also lead to problems in participant compliance due to costs. We may require funding in future studies to mitigate this, as funding limitations have prevented us from employing this approach in this trial. Second, our blinding procedure follows that used in most other tDCS studies, yet recent evidence suggests that this may be insufficient to mitigate the placebo effect. Turi et al. (2019) [18] showed that healthy volunteers were able to distinguish the fade-in, short stimulation, and fade-out method of sham stimulation, described above, from true active stimulation at a level significantly greater than chance. It is not yet known how generalizable these findings are to a population with depression, but there is a need for more research on alternative methods of blinding in tDCS studies.

In conclusion, this trial is a pragmatic randomized double-blinded sham-controlled study that seeks to compare the impact of adjunctive intensive tDCS with left DLPFC positive stimulation or right OFC negative stimulation versus sham tDCS in treating patients with TRD. To our knowledge, this study is the first tDCS protocol of different stimulation targets in this patient population and the first to employ detailed measures of depressive symptoms combined with biomarkers and rs-fMRI data, which will also be used in future analyses. Given the immense health and socioeconomic impacts of TRD, new and effective treatments beyond pharmacotherapy and psychotherapy are needed; tDCS may prove useful in this regard.

## Data Availability

During the ongoing data collection researchers will only have restricted access to the data. After completion of the study, only the PI (WH) and the study coordinator (YC) will have access to the data. Raw data cannot be made openly accessible to the public, due to privacy concerns. After request to the corresponding author, the data, statistical parameters and statistical code will be made accessible. Results will be published in international peer-reviewed journals and will be presented at scientific conferences. We will also report our results to trial summaries. The publication of the results will be independent of the results, whether or not the experimental intervention is effective. Eligibility of authorship will be decided according to the guidelines of the International Committee of Medical Journal Editors (ICMJE).

## Abbreviations

ANOVA: Analysis of variance
BDNF: Brain-derived neurotrophic factor
CGI: Clinical Global Impression Scale
CONSORT: Consolidated Standards of Reporting Trials
DLPFC: Dorsolateral prefrontal cortex
DMC: Data monitoring committee
DSM-5: Diagnostic and Statistical Manual of Mental Disorders, Fifth Edition
DTI: Diffusion tensor imaging
ECT: Electroconvulsive therapy
ELISA: Enzyme-linked immunosorbent assay
GDNF: Glial cell line-derived neurotrophic factor
HAMD-17: Hamilton Depression Rating Scale
ID: Identification document
IL: Interleukin
MADRS: Montgomery and Asberg Depression Rating Scale
MDD: Major depression disorder
MECT: Modified electroconvulsive therapy
MINI: Mini International Neuropsychiatric Interview
MLR: Mixed lymphocyte reaction
NGF: Nerve growth factor
NLR: Neutrophil-to-lymphocyte ratio
ODQ: Oxford Depression Questionnaire
OFC: Orbitofrontal cortex
PI: Principal investigator
PLR: Platelet-to-lymphocyte ratio
rs-fMRI: resting state functional magnetic resonance imaging
SAE: Serious adverse event
SCWT: Stroop Color and Word Test
SD: Standard deviation
SDS: Sheehan Disability Scale
sTNFr1: soluble tumor necrosis factor receptor 1
sTNFr2: soluble tumor necrosis factor receptor 2
tDCS: transcranial direct current stimulation
TNF-α: Tumor necrosis factor- alpha
TRD: Treatment resistant depression
WCST: Wisconsin Card Sorting Test
YMRS: Young Mania Rating Scale

## References

[1] Mrazek DA, Hornberger JC, Altar CA, Degtiar I (2014). A review of the clinical, economic, and societal burden of treatment-resistant depression: 1996-2013. Psychiatr Serv.

[2] Greden JF (2001). The burden of disease for treatment-resistant depression. J Clin Psychiatry.

[3] Souery D, Oswald P, Massat I, Bailer U, Bollen J, Demyttenaere K (2007). Clinical factors associated with treatment resistance in major depressive disorder: results from a European multicenter study. J Clin Psychiatry..

[4] Semkovska M, McLoughlin DM (2010). Objective cognitive performance associated with electroconvulsive therapy for depression: a systematic review and meta-analysis. Biol Psychiatry.

[5] Brunoni AR, Moffa AH, Fregni F, Palm U, Padberg F, Blumberger DM (2016). Transcranial direct current stimulation for acute major depressive episodes: meta-analysis of individual patient data. Br J Psychiatry.

[6] Dell’osso B, Zanoni S, Ferrucci R, Vergari M, Castellano F, D’Urso N (2012). Transcranial direct current stimulation for the outpatient treatment of poor-responder depressed patients. Eur Psychiatry.

[7] Li MS, Du XD, Chu HC, Liao YY, Pan W, Li Z (2019). Delayed effect of bifrontal transcranial direct current stimulation in patients with treatment-resistant depression: a pilot study. BMC Psychiatry.

[8] Blumberger DM, Tran LC, Fitzgerald PB, Hoy KE, Daskalakis ZJ (2012). A randomized double-blind sham-controlled study of transcranial direct current stimulation for treatment-resistant major depression. Front Psychiatry.

[9] Palm U, Schiller C, Fintescu Z, Obermeier M, Keeser D, Reisinger E (2012). Transcranial direct current stimulation in treatment resistant depression: a randomized double-blind, placebo-controlled study. Brain Stimul.

[10] Bennabi D, Nicolier M, Monnin J, Tio G, Pazart L, Vandel P (2015). Pilot study of feasibility of the effect of treatment with tDCS in patients suffering from treatment-resistant depression treated with escitalopram. Clin Neurophysiol.

[11] O'Doherty J, Kringelbach ML, Rolls ET, Hornak J, Andrews C (2001). Abstract reward and punishment representations in the human orbitofrontal cortex. Nat Neurosci.

[12] Guerra-carrillo B, Mackey AP, Bunge SA (2014). Resting-state fMRI: a window into human brain plasticity. Neuroscientist..

[13] Chan A-W, Tetzlaff JM, Altman DG, Laupacis A, Gøtzsche PC, Krleža-Jerić K (2015). SPIRIT 2013 statement: defining standard protocol items for clinical trials. Rev Panam Salud Publica.

[14] Hamilton M (1960). A rating scale for depression. J Neurol Neurosurg Psychiatry.

[15] Seibt O, Brunoni AR, Huang Y, Bikson M (2015). The pursuit of DLPFC: non-neuronavigated methods to target the left dorsolateral prefrontal cortex with symmetric bicephalic transcranial direct current stimulation (tDCS). Brain Stimul..

[16] Cieslik EC, Zilles K, Caspers S, Roski C, Kellermann TS, Jakobs O (2013). Is there "one" DLPFC in cognitive action control? Evidence for heterogeneity from co-activation-based parcellation. Cereb Cortex.

[17] Schulz KF, Altman DG, Moher D, CONSORT Group (2010). CONSORT 2010 statement: updated guidelines for reporting parallel group randomised trials. J Pharmacol Pharmacother.

[18] Turi Z, Csifcsák G, Boayue NM, Aslaksen P, Antal A, Paulus W (2019). Blinding is compromised for transcranial direct current stimulation at 1 mA for 20 min in young healthy adults. Eur J Neurosci.

